# A Case Report of Venous Ulcer Mimicking Cutaneous Leishmaniasis

**DOI:** 10.1002/ccr3.70648

**Published:** 2025-07-16

**Authors:** Alaa Tajeldeen Habeeb Abdallah, Emmanuel Edwar Siddig, Jean Claude Semuto Ngabonziza, Claude Mambo Muvunyi, Ayman Ahmed

**Affiliations:** ^1^ Rufa'a Teaching Hospital Rufaa Sudan; ^2^ Faculty of Medical Laboratory Sciences University of Khartoum Khartoum Sudan; ^3^ Rwanda Biomedical Centre Kigali Rwanda; ^4^ Department of Clinical Biology University of Rwanda Kigali Rwanda; ^5^ Research, Innovation and Data Science Division, Rwanda Biomedical Centre Kigali Rwanda; ^6^ Institute of Endemic Diseases, University of Khartoum Khartoum Sudan

**Keywords:** clinical mimics, leishmaniasis, misdiagnosis, public health, venous ulcer

## Abstract

Venous ulcers can sometimes be difficult to diagnose accurately because they can resemble other skin lesions such as cutaneous leishmaniasis. Here, we present a patient with a venous ulcer that mimics cutaneous leishmaniasis and mycetoma lesions. A 50‐year‐old female patient presented with a nonhealing venous ulcer in her right lower leg, which was suspected to be cutaneous leishmaniasis, ulcerated mycetoma lesions, and misdiagnosed as cutaneous leishmaniasis. She was referred to Rufa'a teaching hospital, Al‐Gezira State, Sudan, where color Doppler sonography results revealed several findings that are consistent with chronic venous insufficiency, including incompetent superficial femoral junction (SFJ) with short reflux among the great saphenous vein (GSV) and incompetent saphenopopliteal junction (SPJ) with short reflux along the small saphenous vein (SSV). Additionally, the examination showed patency and compressibility of both GSV and SSV, as well as the presence of superficial varicose veins. Surgical closure of the fistula was done. This case report highlights the challenges in distinguishing venous ulcers from mycetoma and cutaneous leishmaniasis. The current case emphasizes the importance of considering a comprehensive assessment of the patient's medical history, physical examination, and potentially other diagnostic tests. Collaborating with experienced healthcare providers, such as dermatologists or wound care specialists, may also be beneficial in confirming the diagnosis and developing an appropriate treatment plan.

**Summary**

- Accurate diagnosis of venous ulcers is essential, as they can mimic conditions like cutaneous leishmaniasis.
- A comprehensive evaluation, including medical history, physical examination, and diagnostic tests like Doppler imaging, is crucial to differentiate between various causes and develop an effective treatment plan for effective management.
- Collaboration with specialists can enhance diagnostic accuracy.

## 1 Introduction

The three primary types of ulcers that affect the lower extremities are venous ulcers, arterial ulcers, and neuropathic ulcers [1, 2]. Venous ulcers are the most common type, whereas foot ulcers often stem from arterial insufficiency or neuropathy [2]. Approximately 80% of leg ulcers are attributed to venous disease, while arterial disease accounts for around 10%–25% and can coexist with venous conditions [2]. With an aging population, the incidence of arterial insufficiency may increase. Moreover, about 10%–15% of patients with leg ulcers have coexisting rheumatologic disease, and 5%–12% have diabetes mellitus [2, 3, 4, 5]. Additionally, less common causes such as trauma, pressure, or infectious agents such as buruli ulcer, cutaneous leishmaniasis, and ulcerated mycetoma lesion can lead to leg ulcers [2, 3, 4, 5, 6, 7]. These causes can overlap and occur alongside other medical conditions, as they are not mutually exclusive [8].

The course and prognosis of leg ulcers can differ significantly based on their underlying causes [2]. Venous ulcers are generally less painful and have a lower risk of leading to amputation compared to those caused by arterial insufficiency [2]. However, they remain chronic and may exhibit unpredictable tendencies. The enduring nature of venous ulcers coupled with associated morbidity and financial burden has intensified efforts to develop innovative approaches to enhance healing and improve patient outcomes [2]. A key misconception that needs to be addressed is the pain associated with venous ulcers; recent studies indicate that up to three‐fourths of patients with venous ulcers experience pain, adversely affecting their quality of life. Venous ulcers share overlapping symptoms with cutaneous leishmaniasis, including chronicity, inflammation, pain, and recurrence of ulcers, which can complicate the accuracy of diagnosis. In endemic regions where cutaneous leishmaniasis and atypical mycetoma are prevalent, distinguishing between these conditions becomes even more challenging. Cutaneous leishmaniasis typically presents as irregularly shaped ulcers with raised edges and a characteristic “violaceous” appearance, often accompanied by satellite lesions and local lymphadenopathy. Various serological tests as well as molecular techniques can aid in the diagnosis of CL, and it is advisable to utilize these methods to enhance diagnostic accuracy [9, 10, 11]. In contrast, atypical mycetoma may manifest as firm, painless nodules that can eventually lead to ulceration. The convergence of these symptoms underscores the importance of careful clinical evaluation and differential diagnosis to ensure appropriate treatment for patients presenting with chronic ulcerative conditions.

This communication presents a patient with a venous ulcer that initially mimicked cutaneous leishmaniasis and a mycetoma lesion.

## 2 Case History

A 50‐year‐old female from Al‐Gezira State, presented with recurrent right lower limb swelling, dilated veins, and multiple leg ulcers. She also experienced fever and pain in the affected leg. The wound was previously treated for cutaneous leishmaniasis, but there was no response to the therapy. The patient was administered sodium stibogluconate (SSG) intravenously at a dosage of 20 mg/kg for 30 days and received thermal therapy, as well as paromomycin at 15 mg/kg intramuscularly for 17 days. Her medical history began 15 years ago, shortly after giving birth to her only child. Following delivery, she developed severe pain in her right lower limb and was admitted to the hospital, where she was diagnosed with deep vein thrombosis. Treatment with heparin improved her condition initially, but she continued to experience swelling in her leg over the subsequent years, accompanied by superficially dilated veins. Approximately 10 years ago, the right leg started showing color variations on different sides. Gradually, small lesions developed, causing excruciating pain and elevated body temperature. Over time, these lesions progressed into ulcers of various sizes. The patient had been managing the condition by dressing the ulcers and wearing compression stockings.

Five years ago, she was scheduled for varicose vein surgery; however, the surgery was canceled due to weak veins discovered during the procedure. No other significant findings were noted in her medical history.

## 3 Methods

**FIGURE 1:**
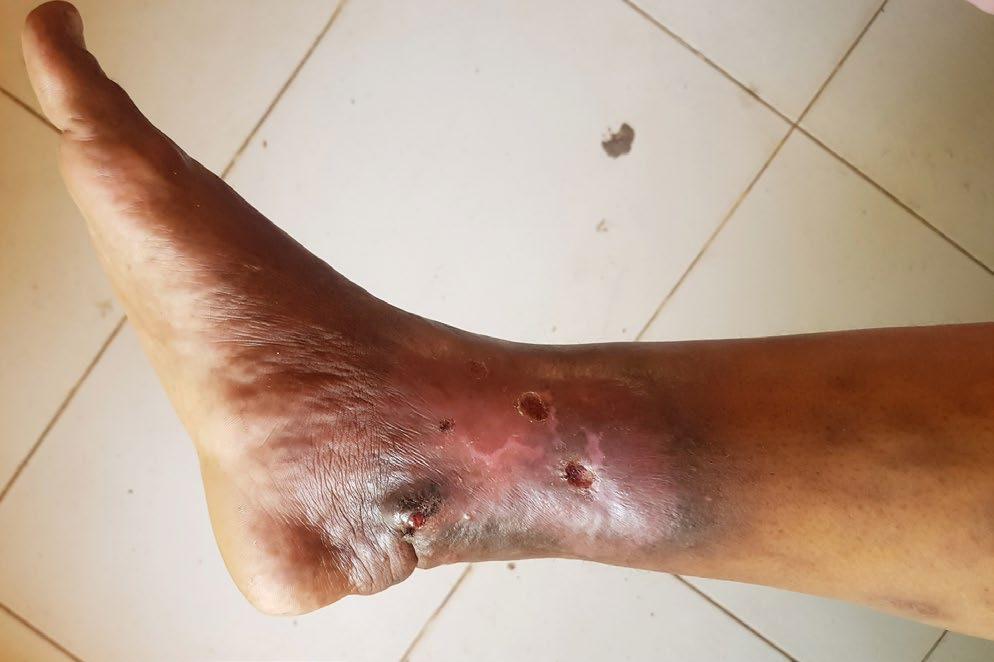
Image illustrating two ulcers present, each measuring approximately 2 × 2 cm, with edges that slope and a red floor.

On examination, the right lower limb exhibited significant swelling compared to the left limb, along with dilated superficial veins below the knee. There was noticeable discoloration in the distal leg, primarily medially over the ankle joint. The area showed varying shades of darkness at the periphery, gradually transitioning into a tight pink to reddish center. Multiple lesions were present, including a 3 × 2 cm dark swelling with a red sore measuring 0.5 cm located above the medial malleolus, and two ulcers measuring approximately 2 × 2 cm each with sloping edges and a red floor (Figure 1). The skin in the affected leg was tough and warm when compared to the other leg. No regional lymphadenopathy was observed, and distal pulsation remained intact. Press‐Imprint cytological smear was performed on the ulcers, which revealed no malignant changes, and the scraping materials were negative for Tuberculosis and Leishmania using molecular assay.

## 4 Conclusion and Results

The patient was diagnosed with chronic varicose veins after color Doppler sonography results revealed several findings that are consistent with chronic venous insufficiency, including incompetent superficial femoral junction (SFJ) with short reflux among the great saphenous vein (GSV), and incompetent saphenopopliteal junction (SPJ) with short reflux along the small saphenous vein (SSV). She was prescribed ceftriaxone injections (1 g twice daily), metronidazole drip (500 mg every 8 h), benzylpenicillin (2 million units every 6 h), and daily wound dressing. She was discharged in a stable condition.

## 5 Discussion

In this communication, we reported on a 50‐year‐old female patient with a history of recurrent right lower leg swelling, dilated veins, and multiple ulcers. This case highlights the challenges in diagnosing and managing chronic venous insufficiency and its complication. Deep vein thrombosis was the initial diagnosis, but the persistence and progression of symptoms suggested an underlying venous pathology. In this case, the patient's persistent symptoms and lack of response to standard treatments for cutaneous leishmaniasis underscore the complexity of her condition. Given that she resides in Al‐Gezira State, a region endemic to cutaneous leishmaniasis, it is understandable that her initial treatment included sodium stibogluconate (SSG), thermal therapy, and paromomycin. However, the absence of healing in her ulcers raises concerns about potential misdiagnosis or treatment resistance. Additionally, her history of deep vein thrombosis and ongoing venous insufficiency suggest that her leg ulcers may not solely originate from leishmaniasis but could also be exacerbated by venous stasis and associated vascular complications. The progression of lesions, along with the presence of recurrent fever and pain, indicates the possibility of an underlying pathology that warrants further investigation. This highlights the importance of reevaluating the diagnosis and considering alternative therapeutic interventions, as conventional treatments may not adequately address the multifactorial nature of her condition.

Diagnosing venous ulcers in regions like Sudan, where endemic diseases—both infectious and noninfectious—are prevalent [12, 13, 14, 15, 16, 17, 18, 19, 20, 21, 22, 23], poses significant challenges. The complexity arises from the fact that leg ulcers can result from a variety of underlying causes, including systemic health issues (Table 1), poor access to healthcare, and the impact of prolonged conflict and instability [2, 7, 24, 25, 26, 27]. It is essential to determine the specific etiology when a patient presents with an ulcer involving the leg because the appropriate treatment may vary depending on the cause.

**TABLE 1:** Causes of leg ulceration.

Diseases	Venous disease
	Arterial disease
	Rheumatoid arthritis
	Diabetes
	Vasculitis
	Scleroderma
	Pyoderma grangrenosum
	Trauma
Infectious agents	Cutaneous leshmaniasis
	Buruli ulcer
	Cutaneous tuberculosis
	Ulcerated mycetoma lesion
Neoplastic	Ulcerating skin cancer

For ulcers on the leg, excluding the foot, the most common cause is chronic venous disease, either alone or in conjunction with other factors that impair healing, such as arterial disease, diabetes, or rheumatoid arthritis [28]. In this particular case, the patient was initially misdiagnosed as having cutaneous leishmaniasis. The endemic nature of cutaneous leishmaniasis in the region, combined with a lack of awareness among general practitioners about other clinical mimics of the condition and limited access to Doppler imaging, likely contributed to this misdiagnosis. As a result, the physician heavily relied on clinical findings alone, without the benefit of additional investigations.

Interestingly, in the presented case, it is commendable that the healthcare team took proactive steps to rule out infectious diseases, such as cutaneous leishmaniasis, mycetoma, and tuberculosis. Molecular testing of the scraping and aspirating materials played a crucial role in excluding these infectious etiologies [29, 30, 31]. This highlights the importance of considering and investigating various possible causes before reaching a final diagnosis.

Furthermore, the use of Doppler scan in this case was instrumental in confirming the diagnosis of chronic venous disease and its associated complications [32]. The Doppler scan likely provided valuable information about the blood flow patterns, venous insufficiency, and potential anatomical abnormalities within the venous system of the affected limb [29]. This imaging modality is particularly helpful in assessing the venous system and its role in the development of leg ulcers.

This case underscores the importance of thorough clinical evaluation and differential diagnosis when addressing leg ulcers, particularly in regions where endemic diseases, such as cutaneous leishmaniasis, complicate the clinical picture. The patient's misdiagnosis and subsequent treatment highlight the necessity of considering all potential underlying causes, including chronic venous insufficiency, especially in patients with a history of deep vein thrombosis. By employing an integrated approach that combines clinical assessment with molecular testing and Doppler imaging, healthcare providers can arrive at more accurate diagnoses and develop effective treatment plans. This case serves as a reminder of the complexities involved in managing chronic ulcerative conditions and the critical need for ongoing education and resources in regions affected by both infectious and noninfectious diseases. It emphasizes that accurate diagnosis is key to improving patient outcomes and enhancing the quality of care provided to individuals suffering from these challenging conditions.

## Author Contributions

**Alaa Tajeldeen Habeeb Abdallah:** conceptualization, data curation, formal analysis, investigation, methodology, validation, visualization, writing – original draft, writing – review and editing. **Emmanuel Edwar Siddig:** conceptualization, data curation, formal analysis, investigation, methodology, validation, visualization, writing – original draft, writing – review and editing. **Jean Claude Semuto Ngabonziza:** investigation, methodology, validation, visualization, writing – original draft, writing – review and editing. **Claude Mambo Muvunyi:** investigation, methodology, supervision, validation, visualization, writing – original draft, writing – review and editing. **Ayman Ahmed:** conceptualization, data curation, investigation, methodology, validation, visualization, writing – original draft, writing – review and editing.

## Ethics Statement

The authors have nothing to report.

## Consent

Written informed consent was obtained from the patients for publication of this case report and any accompanying images.

## Conflicts of Interest

The authors declare no conflicts of interest.

## Data Availability

The data that support the findings of this study are available from the corresponding author upon reasonable request.
